# Impact of Food Origin *Lactiplantibacillus plantarum* Strains on the Human Intestinal Microbiota in an *in vitro* System

**DOI:** 10.3389/fmicb.2022.832513

**Published:** 2022-04-05

**Authors:** Natalia Garcia-Gonzalez, Joan Colom Comas, Hugh M. B. Harris, Conall Strain, Catherine Stanton, Colin Hill, Aldo Corsetti, Cormac G. M. Gahan

**Affiliations:** ^1^Faculty of Bioscience and Technology for Food, Agriculture and Environment, University of Teramo, Teramo, Italy; ^2^School of Microbiology, University College Cork, Cork, Ireland; ^3^APC Microbiome Ireland, University College Cork, Cork, Ireland; ^4^Teagasc Moorepark Food Research Centre, Fermoy, Ireland; ^5^School of Pharmacy, University College Cork, Cork, Ireland

**Keywords:** *ex vivo* model, fecal fermentation, gut microbiota, *Lactiplantibacillus plantarum*, short-chain fatty acid

## Abstract

We have previously isolated and characterized food-dwelling strains of *Lactiplantibacillus* (*Lpb.*) *plantarum* that are consumed naturally as part of the microbiota of table olives and raw milk cheeses. Despite being consumed at relatively high levels, the impact of such strains on the human gut microbiota is currently unclear. In the current study we evaluated the potential impact of food-dominant *Lpb. plantarum* strains on the human gut microbiota using a continuous fecal fermentation system. Daily inoculation of *Lpb. plantarum* strains led to significant, detectable levels in the fecal fermentation system. We examined the impact of the presence of *Lpb. plantarum* on the microbiota derived from two separate donors. For one donor, *Lpb. plantarum* increased alpha diversity and beta diversity. This was reflected in significant alterations in abundance of the unclassified genera, dominated by Enterobacteriaceae_unclass and Ruminococcaceae_unclass. The microbiota of the other donor was relatively unaffected following introduction of the *Lpb. plantarum* strains. Overall, the work describes the response of the human microbiota to the introduction of high levels of food-dominant *Lpb. plantarum* strains and indicates that the response may reflect interindividual differences between donor samples.

## Introduction

The human gastrointestinal tract harbors a highly complex community of microorganisms including bacteria, archaea, protist, fungi, and viruses. This community contributes essential biological functions to the host including the production of vitamins, absorption of ions, protection against pathogens, and enhancement of the immune system (Rousseaux et al., 2007). Host physiology and homeostasis are therefore influenced by this microbial community, the diversity of which can be perturbed by internal and external factors such as age, diet, and disease (Sivieri et al., 2013; Whelan and Quigley, 2013). Dietary microorganisms have the potential to perturb the existing community structure of the gut microbiota in a similar manner to probiotic interventions, but the effects of food-origin strains remain under-researched relative to strains of gastrointestinal origin (Zuo and Ng, 2018).

Live therapeutic (probiotic) interventions have been shown to regulate gastrointestinal barrier function, to increase the adherence of commensal bacteria and to trigger anti-inflammatory responses in the gut (Hill et al., 2014; Plaza-Diaz et al., 2019). Recently, natural food-origin strains, including *Lactiplantibacillus* (*Lpb.*) *plantarum* isolates displaying probiotic properties, have gained greater attention (Derrien and van Hylckama Vlieg, 2015), and studies in mice have indicated some potential health-promoting properties (Park et al., 2017; Heeney et al., 2018). Different strains of this species were able to induce changes in the mouse microbiota (Park et al., 2017; Linninge et al., 2019), improve intestinal barrier function in a clinical trial (Mujagic et al., 2017), and ameliorate colitis-modulating T-cell responses (Kim et al., 2020). However, the impact of consuming high concentration of autochthonous, food-dwelling organisms on the community structure of the human gut microbiota remains to be fully established. *Ex vivo* approaches like fecal fermentations have been used to evaluate changes in colonic microbiota. A significant benefit of this system is that it allows monitoring of alterations to human microbial community structure in real time using a controlled environment. These approaches can therefore be useful as a screening method to test the efficacy of probiotics, prebiotics, and antibiotics in a human-relevant model (Rousseaux et al., 2007; Park et al., 2017). We therefore analyzed the impact of two food-borne strains of *Lpb. plantarum* upon the community structure of human microbiota using timed 16s DNA sequencing in a controlled fecal fermentation system. We have previously shown that these strains, *Lpb. plantarum* C9O4 and LT52, are naturally consumed at high concentrations (∼10^8^ CFU per gram) in table olives (Perpetuini et al., 2020) and cheese (Licitra and Carpino, 2014), respectively, and demonstrate a range of potentially beneficial properties including high adhesion to colonic mucosal cells (Garcia-Gonzalez et al., 2018) and other valuable characteristics (Garcia-Gonzalez et al., 2020; Prete et al., 2020). Here we describe for the first time how such strains survive and impact human microbiota populations.

## Materials and Methods

### Bacterial Strains and Culture Methods

Two *Lpb. plantarum* strains were previously isolated from olives (strain C9O4) and from raw-milk cheese (strain LT52) and were subjected to further study here (Garcia-Gonzalez et al., 2018; Prete et al., 2020). The strains were routinely grown in de Man, Rogosa, and Sharpe (MRS) broth and agar (1%), and when counts were required, serial dilutions were done in phosphate-buffered saline (PBS). To track their survival in complex fecal samples, rifampicin-resistant derivatives of these strains were selected using MRS agar plates supplemented with increasing concentrations of the antibiotic to a maximum of 50 μg/ml (Sigma). Single colonies were selected from these plates and inoculated into fresh MRS broth producing the strains *Lpb. plantarum* C9O4 Rif*^R^* and *Lpb. plantarum* LT52 Rif*^R^*. Neither strain demonstrated any deficiency in growth relative to Rif*^S^* wild-type strains across a range of *in vitro* growth conditions (data not shown). These cultures were preserved at −80°C in 20% glycerol.

### *In vitro* Continuous Fecal Fermentations

Fecal samples were collected at one time point from two different individuals, one apparently healthy adult and an inflammatory bowel disease (IBD) patient. The healthy sample, 956, was collected from a healthy individual aged 29 years; the same collection procedure was used for the IBD002 sample, obtained from a patient who was 50 years old. The fecal samples were diluted (1:10) in anaerobic basal broth (ABB, Sigma-Aldrich) supplemented with 15% glycerol and homogenized in a stomacher for 2 min. The resulting fecal slurries had a final concentration of 25% (w/v) and were used to inoculate the vessels. Fermenter vessels were autoclaved and aseptically filled with 200 ml of modified yeast extract, casitone and fatty acid – glucose, starch, and cellobiose medium (YCFA-GSCM) medium [casitone (10 g/l), yeast extract (2.5 g/l), NaHCO_3_ (4 g/l), L-cysteine (1 g/l), glucose (2 g/l), cellobiose (2 g/l), maltose (2 g/l), soluble starch (2 g/l), K_2_HPO_4_ (4.5 g/l), KH_2_PO_4_ (4.5 g/l), NaCl (9 g/l), (NH_4_)_2_SO_4_ (9 g/l), MgSO_4_ 1 M (1 mM), CaCl_2_ 1 M (1 mM), Haemin 5,000 × concentrate (0.2 ml/l), Resazurin 1,000 × concentrate (1 ml/l), and vitamins 1,000 × concentrate (1 ml/l)] (Li et al., 2018). Prior to the inoculation with 12.5 ml of the fecal slurry (1:10 w/w), the media was allowed to adapt to environmental conditions mimicking those of the colon, 0.1% dissolved oxygen, pH 6.8, and 37°C. Once inoculated, the microbial community was left to stabilize over a period of 24 h. After that, the treatment vessel was inoculated daily with a combination of *Lpb. plantarum* C9O4-Rif*^R^* and *Lpb. plantarum* LT52-Rif*^R^* in equal amounts to a final concentration of 10^7^ CFU/ml, while the other vessel remained as control. The inoculation with *Lpb. plantarum* strains (10^7^ CFU/ml/day) was performed for three consecutive days after the o/n stabilization of the slurries (D0, D1, and D2). Prior to each inoculation with *Lpb. plantarum*, samples were collected at 0, 24, 48, and 72 h, corresponding to D0, D1, D2, and D3, to check bacterial counts and short-chain fatty acid (SCFA) and microbiota composition. For both types of samples, healthy and IBD donors, the fermentations were carried out with and without the addition of *Lpb. plantarum* strains (daily inoculum of 10^7^ CFU/ml) (Supplementary Figure 1B). Experiments were carried out in triplicate for the IBD sample and in quadruplicate for the healthy sample. All samples, except those used for the daily enumeration of *Lpb. plantarum*, were blast frozen using liquid nitrogen and stored at −80°C until use.

### DNA Extraction From Fecal Samples and 16S Metagenomic Library Preparation

DNA was extracted from 2 ml fecal sample using the QIAamp DNA Stool Mini Kit (Qiagen, Hilden, Germany) according to the manufacturer’s protocol. DNA yield was quantified using the Invitrogen Qubit 4 fluorometer (Thermo Fisher Scientific, Waltham, MA, United States). Sequencing libraries of the V3–V4 region were prepared according to the Illumina MiSeq system instructions (F:5′TCGTCGGCAGCGTCAGATGTGTATAAGAGACAGCCT ACGGGNGGCWGCAG3′; R:5′GTCTCGTGGGCTCGGAGATG TGTATAAGAGACAGGACTACHVGGGTATCTAATCC3′). In brief, the V3 and V4 regions of the 16S bacterial rRNA gene were amplified using a two-step polymerase chain reaction (PCR) protocol with V3 and V4 region primers for the first PCR and Nextera XT index primers for the second PCR. Amplicons were cleaned using AMPure XP magnetic beads, and then, Illumina sequencing adapters and dual-index barcodes were added to each amplicon. The library concentrations were assessed with a Qubit dsDNA assay kit (Thermo Fisher Scientific, Waltham, MA, United States). The quality of the library was checked by running 1 μl on a Bioanalyzer DNA1000 chip (Agilent, Santa Clara, CA, United States) to verify the amplification and peak sizes.

### Short-Chain Fatty Acid Quantification

External standards diluted in acidic Mili-Q water (Mili-Q water and 36.5–38% hydrochloric acid) were prepared from stock solutions of acetate, propionate, isobutyrate, butyrate, isovalerate, and valerate (Sigma-Aldrich, St. Louis, MO, United States). In addition, an internal stock of 2-ethylbutyric acid and formic acid was prepared in acidic Milli-Q water (10 mM). A seven-point standard curve was generated [SCFA 0.1, 0.5, 1, 2, 4, 8, and 10 mM; branched-chain fatty acids (BCFA) 0.01, 0.05, 0.1, 0.2, 0.4, 0.8, and 1 mM] in acidic Milli-Q water with 1 mM internal standard.

Fecal samples were filtered with 0.22 mM syringe filters (Corning, NY, United States) and were mixed with internal standard in duplicates. The samples were homogenized briefly and centrifuged at 15,000 rpm for 3 min. The supernatant was transferred to 250 μl inserts (Agilent) placed in amber glass GC vials (2 ml, Agilent, Santa Clara, CA, United States) and sealed with silicone/polytetrafluorethylene (PTFE) screw caps (Agilent, Santa Clara, CA, United States). Standards and samples were analyzed by gas chromatography flame ionization detection (GC-FID) using a Varian 3800 GC system, fitted with an Agilent DB-FFAP column (30 ml × 0.32 mm ID × 0.25 μm df; Phenomenex) and a flame ionization detector with a CP-8400 auto-sampler. Helium was employed as the carrier gas at an initial flow rate of 1.3 ml/min. The oven temperature was initially maintained at 50°C for 30 s. Then, it was raised to 140°C at a rate of 10°C/min and held for 30 s. The last increase was done at a rate of 20°C/min until 240°C was reached and held for 5 min. This summed a total run time of 20 min. The detector and the injection port were kept at 300 and 240°C, respectively. A splitless injection of 0.2 μl was carried out for each sample or standard using a 10 μl syringe (Agilent, Santa Clara, CA, United States) installed to a CP-8400 auto-sampler (Varian). Varian Star Chromatography Workstation version 6.0 software was used for peak integration. Vials containing 1,800 μl of water were run between each sample to check for any potential carryover. Standards were included in each run to maintain the calibration.

### Bioinformatics and Statistical Analysis

A total of 56 samples were sequenced by Eurofins giving paired-end reads of length 301 bp with a mean read number of 44,341 (min = 22,375; max = 79,521). DADA2 (Callahan et al., 2016) was used to trim reads by truncation of forward and reverse reads to 250 and 230, respectively: truncLen = c (250,230). Reads were de-replicated using DADA2 (function: derepFastq) followed by error correction of reads (function: dada) and merging of forward and reverse reads (function: mergePairs). Usearch (v8.1) (Edgar, 2010) was used to remove reads lower than 439 bp and greater than 467 bp and to remove chimeric sequences using UCHIME and the RDP ‘‘Gold’’ database^1^. Reads were classified using mothur (v.1.39.5; function classify.seqs) (Schloss et al., 2009) with its accompanying subset of the Ribosomal Database Project (RDP) database (version 11.5) at a confidence cutoff of 80%. R (v3.6.1; R) (R Core Team, 2016), was used to produce all figures and statistics based on 16S rRNA gene sequencing results: Taxon read counts from phylum to genus level were normalized to percentage data; alpha diversity was calculated for observed species (sequence variant counts from DADA2) and Shannon index; beta diversity was calculated for the Bray–Curtis index using the vegan package (v2.5-5; function vegdist) (Oksanen et al., 2019), visualized using the package made4 (function s.class) (Culhane et al., 2005), and statistical significance for separation of groups was tested using analysis of variance for distance matrices (function adonis in vegan). Significant differences across groups for alpha diversity were tested using the *t*-test in a pairwise manner followed by Benjamini–Hochberg correction of *p*-values. For genus-level statistics, ANOVA was used to test for significance across groups in each genus with a mean ≥ 1%, and all genera with *p* < 0.05 were subject to *post hoc t*-tests to look for pair-wise differences, followed by Benjamini–Hochberg correction of *p*-values.

## Results

### Persistence of Introduced Lactobacilli in the Gut Microbiota Environment

Both strains of *Lpb. plantarum* were combined in equal amounts and introduced into an established intestinal microbiota growing in continuous fermentation. In initial experiments, a single inoculation, *Lpb. plantarum* did not remain at high levels, going from an initial 4.96 × 10^6^ to a final 4.26 × 10^4^ CFU/ml after 72 h (Supplementary Figure 1A). As many probiotics are advised to be taken daily, a test emulating these conditions was subsequently conducted. In this case, *Lpb. plantarum* strains were inoculated every 24 h to evaluate the persistence of the introduced strain in the gut environment.

A single inoculation of the strains was not sufficient to establish *Lpb. plantarum* levels over time. This is potentially because of competitive exclusion exerted by the commensal bacteria present in the gut community (Derrien and van Hylckama Vlieg, 2015). Alternatively, the bacterium may have been washed out of the system at a greater rate than the growth rate in the microbial community. However, when bacteria were inoculated daily, food-borne *Lpb. plantarum* strains were capable of transiently persisting in the community, remaining at high levels for the 3-day duration of the experiment in the artificial gut environment (Supplementary Figure 1A).

### Alpha and Beta Diversity in Model Human Fecal Microbiota Show Significant Changes After Intervention With Lactiplantibacillus Strains

To investigate the impact of the introduction of the *Lpb. plantarum* strains on microbiota composition, three separate and independent experiments were conducted on subsamples of fecal samples collected from one healthy volunteer and an IBD patient. Our rationale was to test two different sample sources with very different microbiotas and to determine how *Lpb. plantarum* strains might impact these communities. We appreciate that the sample size is too small to draw any conclusions based upon microbial communities from patients with IBD. In every case, two fermentations were carried out simultaneously, in which one of the vessels was inoculated with the mix of *Lpb. plantarum* strains while the other remained as an un-inoculated control. Based upon our previous initial studies (Supplementary Figure 1A), the inoculation with *Lpb. plantarum* strains was performed consecutively three times after the overnight stabilization of the slurries.

**FIGURE 1:**
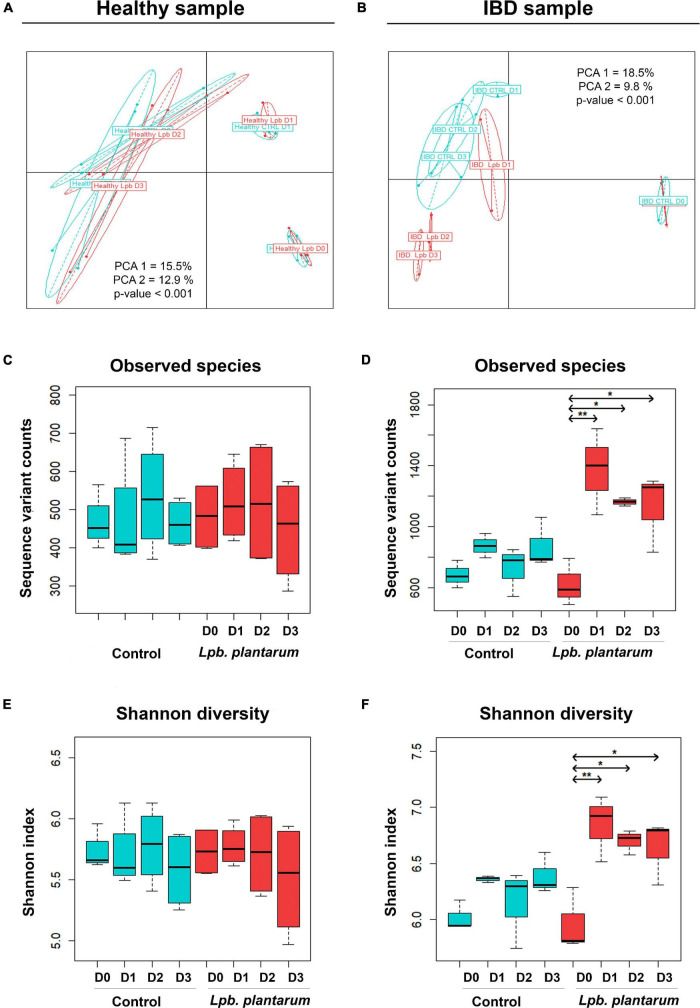
Bray–Curtis PCoA of *Lpb. plantarum* probiotic effect on model gut microbial β-diversity of a healthy individual **(A)** and an IBD patient **(B)**. The Bray–Curtis index was used on sequence variant counts (from DADA2), and groups represent samples taken over a period of 4 days (from D0 to D3); for the control (blue) and *Lpb. plantarum*-inoculated (red) intestinal microbiota. PCA1 and PCA2 represent the percent of variation explained by principal coordinate axes 1 and 2, respectively. The *p*-value is the result of an ANOVA test on a Bray–Curtis distance matrix. *Lpb. plantarum* effect on model gut microbial diversity of a healthy individual **(C**,**E)** and an IBD patient **(D,F)**. Alpha diversity is based on the number of observed species **(C,D)** and Shannon index **(E,F)**. Box plots show diversity over a period of 4 days (from D0 to D3). Experiments were carried out in triplicate with the IBD sample and in quadruplicate with the healthy sample. *p < 0.05, **p < 0.01.

To evaluate the differences in beta diversity of the model microbial communities both with and without introduced *Lpb. plantarum*, we conducted principal coordinate analysis (PCoA) showing sample relatedness using Bray–Curtis dissimilarity. The general composition of both healthy (956) and IBD (IBD002) samples shifted over time regardless of the presence of the introduced lactobacilli (Figure 1; *p* < 0.001). Samples from the *Lpb. plantarum*-inoculated and un-inoculated fermentations from the healthy donor strongly clustered together by time point (Figure 1A), while in the IBD002 microbiota the inoculation of *Lpb. plantarum* strains showed considerable separation from the control group (Figure 1B). Following the introduction of the *Lpb. plantarum* strains, the general microbial composition of the IBD002 sample started to diverge from the control un-inoculated community, showing a gradual change in diversity which increased over time (Figure 1B; *p* < 0.001). While the separation of time points in both 956 and IBD002 microbiota shows a change of general microbial composition over time, the additional separation of the inoculated group in the IBD002 microbiota indicates that the presence of the *Lpb. plantarum* strains is also driving microbial change in this particular community.

Alpha diversity was used to evaluate the abundance and evenness of species in the model gut communities. Shannon index and number of observed species were used to measure alpha diversity (Figures 1C–F). In the case of the IBD002 microbiota, the inclusion of the *Lpb. plantarum* strains significantly enriched the number of species observed in the community, increasing the number of observed species and Shannon index for all time points sampled (Figures 1D,F; *p* < 0.05 and *p* < 0.01). The alpha diversity remained unaltered for the gut microbiota of the healthy individual during the whole treatment with the *Lpb. plantarum* strains (Figures 1C,E).

### Compositional Differences at Phylum, Family, and Genus Levels

**FIGURE 2:**
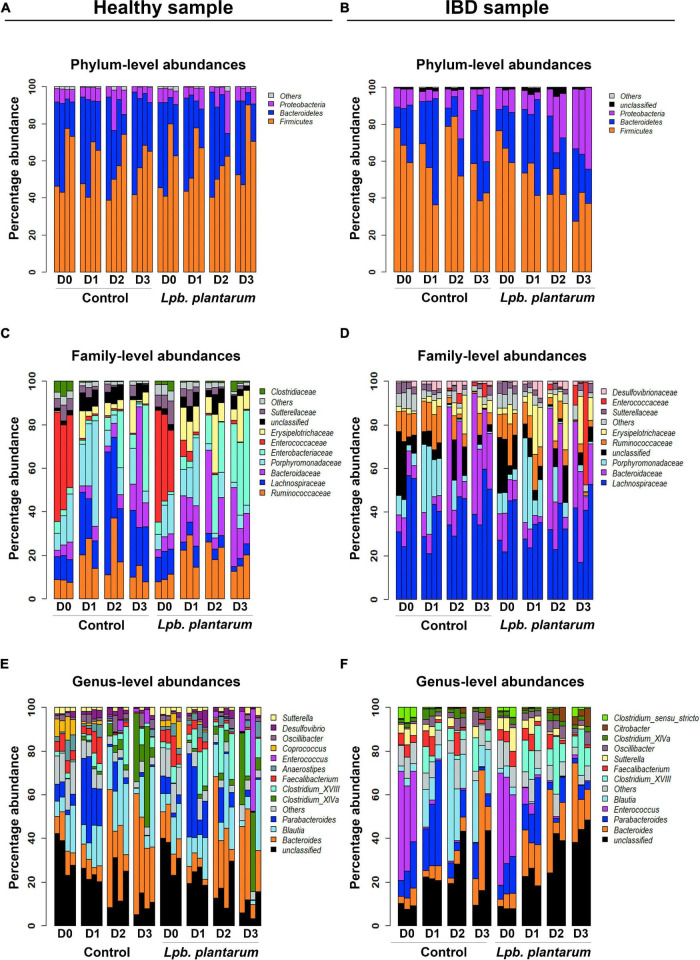
*Lpb. plantarum* effect on model gut microbial relative abundances of a healthy individual and an IBD patient. Relative abundances are presented at phylum **(A,B)**, family **(C,D)** and genus **(E,F)** levels, with and without treatment of the *Lpb. plantarum* inoculum. The “Others” category is a composite group of all low-abundance taxa with a mean less than 1%. The “Unclassified” category represents all reads with a confidence score less than 80% at a given taxon level based on classification with mothur and the RDP database (see section “Materials and Methods”). For each panel, taxa are ordered bottom to top based on mean percent abundance across replicates.

The relative abundances of taxonomic groups from phylum, family, and genus levels in both the 956 sample and the IBD002 sample were characterized to observe the changes induced by the intervention. Analysis using the 16S rRNA gene counts normalized to percentage data indicated that the healthy microbiota composition was dominated by Bacteroidetes, Firmicutes, and Proteobacteria, through all time points of the fermentation (Figure 2A). In all datasets, even in fermenter reactions where *Lpb. plantarum* was added, the *Lactobacillus* genus is present at a relative abundance well below 1% so it was included in the “Others” category. Supporting the results for alpha and beta diversity, the introduction of the *Lactiplantibacillus* strains did not induce any significant changes in the relative abundances of genera in the model healthy microbiota (Supplementary Table 1). At family level there was a sharp spike in abundance of Porphyromonadaceae at D1 for both control and inoculated groups, while Bacteroidaceae increased considerably at D2 and D3. *Erysipelotrichaceae* is also present in noticeably higher abundance from D1–D3 when the *Lpb. plantarum* inoculum is present (Figure 2C). At genus level there was a trend toward an increase in *Clostridium XVIII* and *Enterococcus* when the *Lpb. plantarum* strains were present (Figure 2E). A few genera did significantly increase or decrease over the time points, and the trend was the same for both the healthy (956) and IBD002 microbiota. Parabacteroides increased considerably from D0 to D1, while Clostridium XIVa increased from D2 to D3; both *Coprococcus* and *Faecalibacterium* decreased over time, particularly on D2 and D3 (Figure 2E and Supplementary Table 1; *p* < 0.05).

Similar to the microbiota from the healthy (956) donor, the IBD002 sample was mainly dominated by the phyla Bacteroidetes, Firmicutes, and Proteobacteria (Figure 2B). At the family level, *Enterobacteriaceae* is the main group increasing in the presence of the *Lpb. plantarum* strains, whereas the *Lachnospiraceae* shows a considerable reduction. *Enterococcaceae* is abundant at D0 for both control and inoculated groups but decreased considerably over time (Figure 2D). When analyzing the data at the genus level, both control and treatment IBD002 microbiota had a significant reduction in the proportion of *Enteroccocus* after 24 h regardless of the presence of *Lpb. plantarum* strains, while *Blautia* increased significantly in the control group only (Figure 2F and Supplementary Table 2; *p* < 0.05). It is interesting to note that unclassified genera decreased over time in the healthy (956) donor sample yet increased in the IBD002 sample, regardless of the presence or absence of *Lpb. plantarum*. This unclassified category is a concatenated group of taxa, some of which received a classification at levels higher than genus. For this reason, Supplementary Figure 2 shows the unclassified category separated into its constituent taxon sub-categories for both healthy and IBD002 samples. Here, each unclassified sub-category is labeled as an unclassified taxon belonging to the lowest taxon level for which it was classified (e.g., Lachnospiraceae_unclass is an unclassified genus, or perhaps several genera, from the family *Lachnospiraceae*). The unclassified sub-categories are very different between healthy and IBD, with Lachnospiraceae_unclass being abundant in all healthy replicates and a dominance of Bacteroidetes_unclass in most replicates at D0, while the IBD sample is dominated by Enterobacteriaceae_unclass and Ruminococcaceae_unclass (Supplementary Figure 2).

### Differences in Microbiota Were Not Correlated With Short-Chain Fatty Acid Production

Although our results demonstrated changes in abundance of specific bacterial taxa as a consequence of the inclusion of *Lpb. plantarum* strains in the IBD002 microbiota, the mechanisms behind this modulation are unknown. Considering that the fermentation vessel is a closed system, we hypothesized that end-products of bacterial metabolism could be responsible for driving changes in the microbiota composition in the IBD002 microbiota. Recent reports have indicated a role for SCFAs as key modulators linking gut microbiota, nutrition, and host physiology (Ríos-Covián et al., 2016). Since major changes in microbiota were only observed in the IBD patient, we analyzed for the IBD fecal sample, whether these changes were due to the SCFA or branched-chain SCFA (BSCFA) production. A decrease of the total SCFA was observed in both vessels, inoculated and control, mainly driven by lower levels in acetate and propionate. Levels of butyrate remained stable during the whole fermentation, while valerate increased. However, none of these changes seemed to be due to the inclusion of the *Lpb. plantarum* strains since the trend in both vessels appeared to be the same. Regarding BSCFA, the inclusion of *Lpb. plantarum* strains led to a significantly lower concentration at 72 h (D2) after inoculation. Levels of isobutyrate decreased in the inoculated vessel during the whole fermentation, being significant at 72 h of fermentation (Supplementary Figure 3).

## Discussion

Live therapeutic lactic acid bacteria (probiotics) have been studied extensively for their ability to influence microbiome community structure and host responses with studies particularly focusing upon strains of human or animal origin (Derrien and van Hylckama Vlieg, 2015; Plaza-Diaz et al., 2019). We have previously characterized food-dwelling *Lpb. plantarum* strains that are likely to be consumed at very high levels (∼10^7^–10^8^ CFU/g) in foods such as table olives and raw milk cheeses (Licitra and Carpino, 2014; Perpetuini et al., 2020). Here we used an *ex vivo* fermentation model mimicking the colonic microbiota to study the effect of a combination of two *Lpb. plantarum* strains on the microbial composition and short-chain fatty acid production in microbiotas donated by either a healthy adult (sample 956) or an individual with IBD (IBD002) in order to ascertain the impact of our strains upon microbial communities from two very different donor sources. Evidence suggests that probiotic bacteria normally do not persistently colonize the gastrointestinal tract and need to be ingested daily to maintain their levels in the gut (Derrien and van Hylckama Vlieg, 2015). Indeed, we noted a continuous decline in the levels of *Lpb. plantarum* strains following a single inoculation into our complex model microbiota system. Therefore, a daily inoculation of the strains was implemented to ensure their presence at high levels throughout the whole fermentation.

The inclusion of the *Lpb. plantarum* stains in our system increased alpha diversity in the fermenter seeded with the IBD002 microbiota, with an increase in the number of observed species and Shannon index and also increased beta diversity in this system. However, the same strains had no effect on the alpha-diversity or beta-diversity of the microbiota derived from the healthy 956 sample. The variation in probiotic effects across different subjects has been previously reported in several intervention studies (Costa et al., 2014; Wang et al., 2014), a phenomenon that is likely to be associated with the community structure of the individual’s microbiota (Costa et al., 2014; Zmora et al., 2018). We recognize that our study is not sufficiently powered to make conclusions about impacts upon the IBD microbiota *per se* but demonstrates differing responses to *Lpb. plantarum* interventions in the microbiotas from two individual donors. Further studies will be necessary to document alterations to community structures across greater numbers of individuals.

Using 16s rRNA gene sequencing, we determined the precise alterations to microbial community structure that occurred in the IBD002 microbial community following introduction of *Lpb. plantarum* strains. Previous studies have examined gut microbial changes induced by the presence of *Lpb. plantarum* probiotics in murine colitis models, with apparent variations in Lactobacillales, Clostridiales, Bifidobacteriales, Erysipelotrichales, Bacillales, Verrucomicrobiales, and Enterobacteriales (Park et al., 2013; Zhang et al., 2019). The results from our modeling of human gut microbiota populations agreed with Zhang et al. (2019) who observed a decrease in Firmicutes and an increased presence of unclassified bacteria following introduction of *Lpb. plantarum* in a murine colitis model (Zhang et al., 2019). It is interesting to note significant alterations to unclassified genera in our human microbiota model system (outlined in detail in Supplementary Figure 2), which will form the basis for further functional studies.

These changes in microbial composition in the IBD002 fermenter system may be a consequence of the impact of *Lpb. plantarum* metabolism upon the particular microbial community in this sample. Strains of this species are known to produce SCFAs as secondary metabolites (Botta et al., 2017). No major changes were observed in the SCFA profiles when *Lpb. plantarum* strains were included in the community; however, SCFA could be consumed by other members of the gut microbiota as a substrate or source of energy (Ríos-Covián et al., 2016). For instance, a reduction in isobutyrate has been related to an increased abundance of the genus *Ruminococcus* (*p* < 0.05), which is known to require this compound for its growth (Bernalier et al., 1996). The continuous reduction in the presence of Clostridia when the *Lpb. plantarum* strains were included in the community may be the cause of the reduction in BSCFA and more specifically in isobutyrate. This trend has been previously reported by Rehman et al. (2012) who observed a similar reduction in BSCFA after inclusion of the commercial probiotic formulation #VSL3 (Rehman et al., 2012).

## Conclusion

In conclusion, we used an *in vitro* fermentation model of gut microbiota to characterize the impact of food-origin *Lpb. plantarum* strains on microbiota composition. Our approach represents a human-relevant system which is also an ethical alternative to the use of animals. The overall results showed a modulation of the IBD002 microbiota by the presence of two food-borne *Lpb. plantarum* strains while the microbiota from a different donor was more resistant to change. To our knowledge, it is the first time that the effect of food-associated *Lpb. plantarum* strains have been analyzed in a human model fermenter system, and the work is part of a larger series of experiments from our group to characterize such strains which are likely to be consumed at high levels as part of a typical Mediterranean diet. Further studies with a much larger cohort of donors will be necessary to investigate the interindividual nature of the response and potential impact upon microbiotas from individuals with IBD.

## Data Availability Statement

The datasets presented in this study can be found in online repositories. The names of the repository/repositories and accession number(s) can be found below: https://www.ncbi.nlm.nih.gov/, PRJNA685140.

## Ethics Statement

The studies involving human participants were reviewed and approved by Fecal samples were collected from consenting volunteers according to study protocol APC055, approved by the Cork Research Ethics Committee (CREC). The patients/participants provided their written informed consent to participate in this study.

## Author Contributions

AC, CG, CH, and CaS conceived, designed, and supervised the experiments and corrected the manuscript. NG-G, JC, and CoS performed the experiments. NG-G, JC, and HH analyzed the data, discussed the results, and drafted the manuscript. All authors read and approved the final manuscript.

## Conflict of Interest

The authors declare that the research was conducted in the absence of any commercial or financial relationships that could be construed as a potential conflict of interest.

## Publisher’s Note

All claims expressed in this article are solely those of the authors and do not necessarily represent those of their affiliated organizations, or those of the publisher, the editors and the reviewers. Any product that may be evaluated in this article, or claim that may be made by its manufacturer, is not guaranteed or endorsed by the publisher.

## Footnotes

1
https://drive5.com/uchime/gold.fa
