# Supplementary figures and images for: Impact of Food Origin Lactiplantibacillus plantarum Strains on the Human Intestinal Microbiota in an in vitro System
Source: Front Microbiol. 2022 Apr 5;13:832513. doi: 10.3389/fmicb.2022.832513 (PMC9016340; doi:10.3389/fmicb.2022.832513)

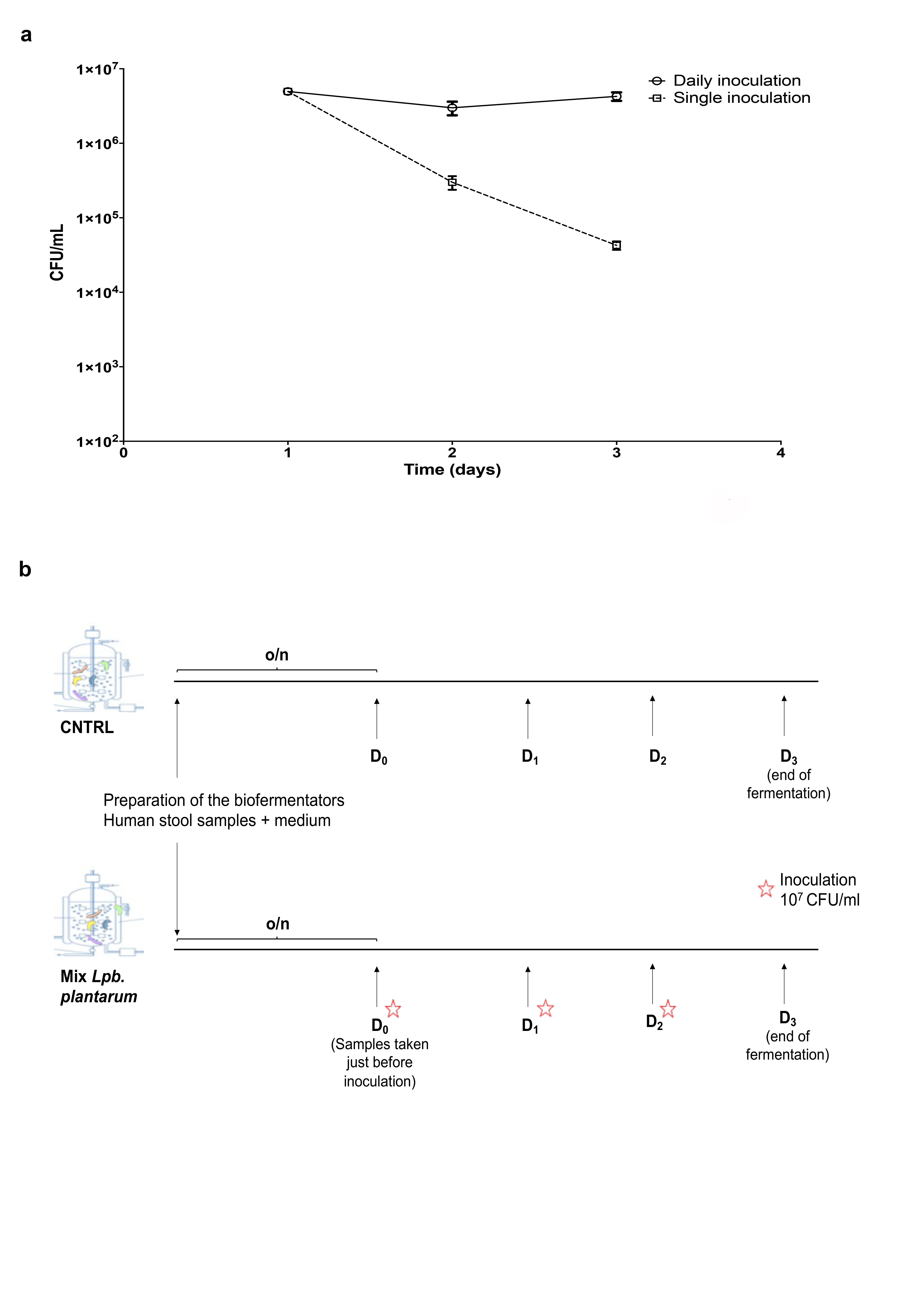

Supplement: Supplementary file 1 [file Image_1.jpeg]

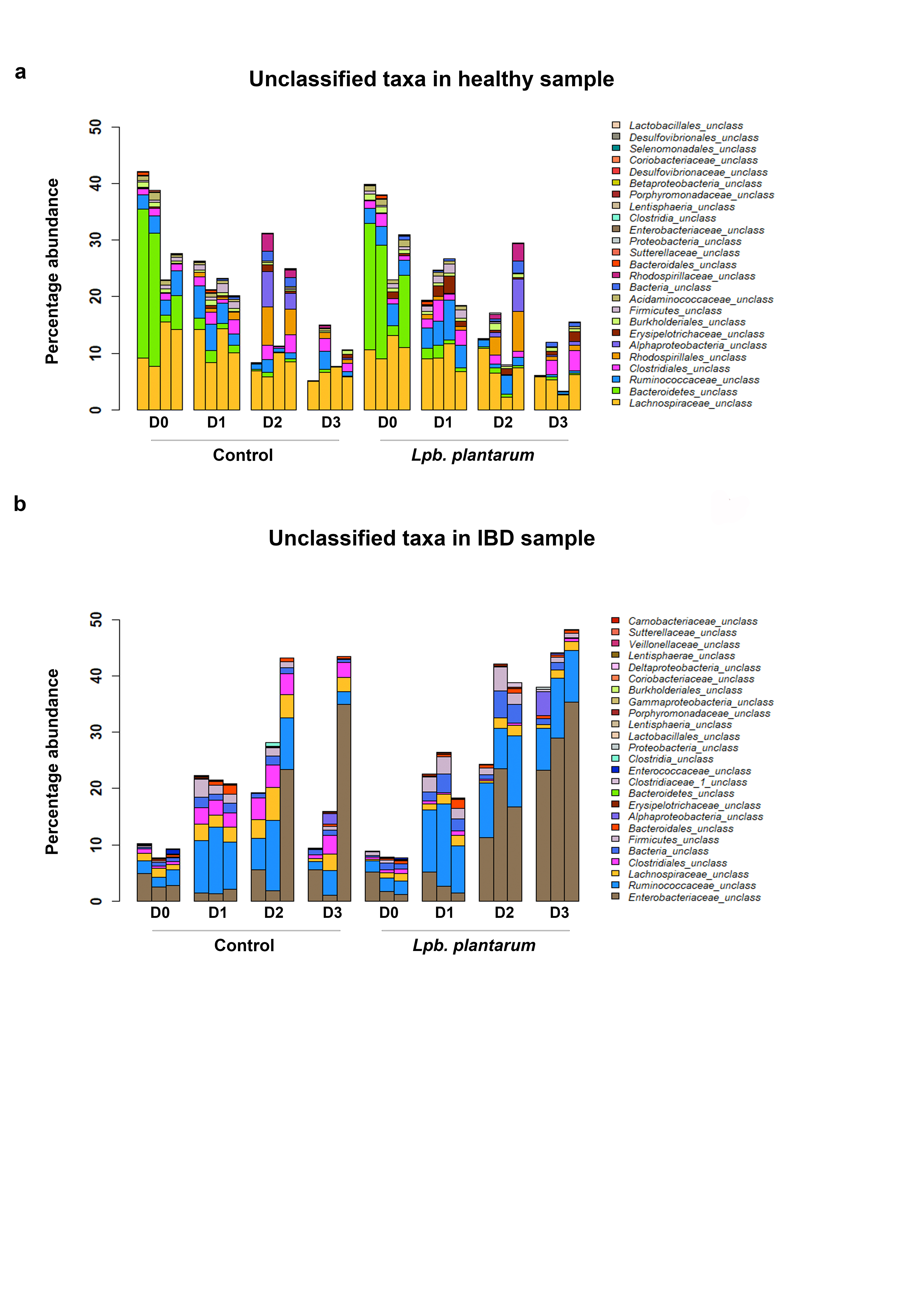

Supplement: Supplementary file 2 [file Image_2.jpeg]

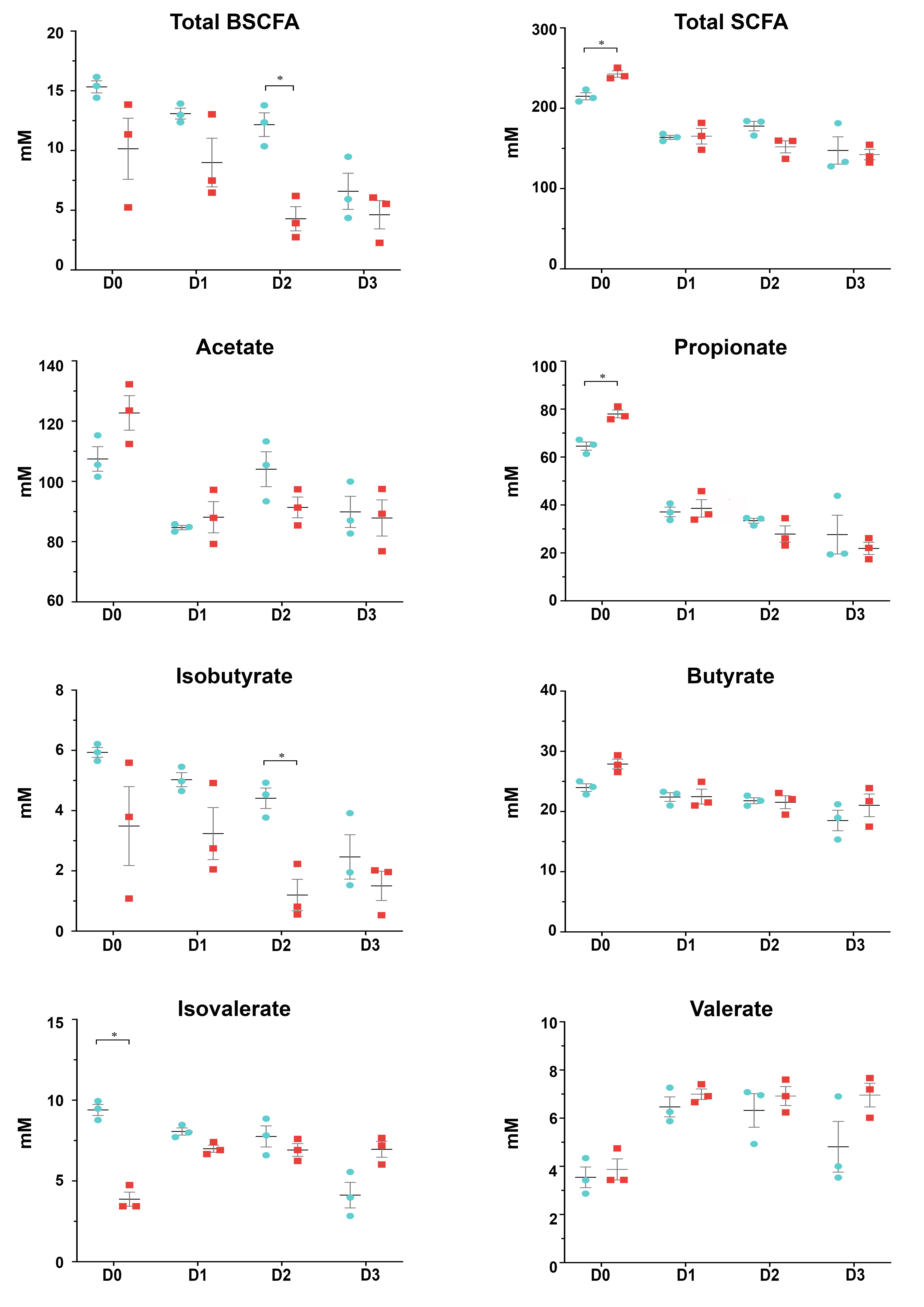

Supplement: Supplementary file 3 [file Image_3.jpeg]
