# Supplementary material for: Impact of Food Origin Lactiplantibacillus plantarum Strains on the Human Intestinal Microbiota in an in vitro System
Source: Front Microbiol. 2022 Apr 5;13:832513. doi: 10.3389/fmicb.2022.832513 (PMC9016340; doi:10.3389/fmicb.2022.832513)
Supplement: Supplementary file 7 [file Data_Sheet_1.docx]

Supplementary Material

# Data availability statement

All raw sequence reads have been deposited in the National Center for Biotechnology Information (NCBI) under the Bioproject accession number PRJNA685140.

# Supplementary Tables and Figures

## Supplementary Figures

**Figure S1. (a) Persistence of the *Lpb. plantarum* strains in the gut microbiota environment after a single and daily inoculation for three days (b) Scheme of the experimental design.** Two vessels inoculated with the same fecal sample (IBD or healthy donor sample) were maintained for four days (D0 – D3) without (control) or with a daily inclusion (10^7^ CFU/ml) of two *Lpb. plantarum* strains. Analyses were performed with samples taken before each inoculation, blast frozen using liquid nitrogen and stored at -80 °C until their use.

**Figure S2. *Lpb. plantarum* effect on model gut microbial relative abundances of a healthy individual and an IBD patient for unclassified sub-categories.** Relative abundances are presented for each unclassified sub-category, with and without treatment of the *Lpb. plantarum* inoculum. The height of each bar reflects the total % abundance of the unclassified category for the same replicate in **Figure 2** (**e - f**). For each panel, taxa are ordered bottom to top based on mean % abundance across replicates.

**Figure S3. *Lpb. plantarum* impact on short-chain fatty acid composition of an IBD patient.** Control un-inoculated is represented in blue, while *Lpb. plantarum*-inoculated is represented in red (n=3). Statistical analysis was conducted using One-way ANOVA and Bonferroni’s Multiple Comparison. Error bars represent standard deviation (SD), * p<0.05

## SupplementaryTables

**Table S1. Tests for significant differences at genus level across all groups in the healthy individual.** All genera with a mean abundance of 1 % or greater and with p<0.05 after ANOVA are included as individual excel sheets. Column one shows the two groups being compared, columns two and three show the mean abundance (%) of the two groups and column 3 shows the adjusted p-value (Benjamini-Hochberg). For column one, S_CNTR = Healthy control and S_Mix = Healthy *Lpb. plantarum* inoculated.

**Table S2. Tests for significant differences at genus level across all groups in the IBD patient.** All genera with a mean abundance of 1% or greater and with p<0.05 after ANOVA are included as individual excel sheets. Column one shows the two groups being compared, columns two and three show the mean abundance (%) of the two groups and column 3 shows the adjusted p-value (Benjamini-Hochberg). For column one, IBD_CNTR = IBD control and IBD_Mix = IBD *Lpb. plantarum* inoculated.
